# Valproate utilisation trends among women of childbearing potential in Ireland between 2014 and 2019: A drug utilisation study using interrupted time series

**DOI:** 10.1002/pds.5427

**Published:** 2022-03-31

**Authors:** John E. Hughes, Niamh Buckley, Yvonne Looney, Sinead Curran, Maeve Mullooly, Kathleen Bennett

**Affiliations:** ^1^ Division of Population Health Sciences Royal College of Surgeons in Ireland Dublin Ireland; ^2^ Health Products Regulatory Authority Kevin O'Malley House, Earlsfort Centre, Earlsfort Terrace Dublin 2 Ireland; ^3^ Data Science Centre, Division of Population Health Sciences Royal College of Surgeons in Ireland Dublin Ireland

**Keywords:** European medicines agency, interrupted time series, pregnancy prevention programme, sodium valproate, teratogenicity

## Abstract

**Purpose:**

This study aimed to examine trends in valproate use among women of childbearing potential (WCBP) aged 16–44 years in Ireland following two European‐directed regulatory interventions in December 2014 and April 2018.

**Methods:**

This was a repeated cross‐sectional study using monthly national pharmacy claims data, to examine trend changes in the prevalence of valproate use among WCBP pre and post two separate regulatory events in December 2014 and April 2018. Annual population estimates from the Central Statistics Office were used to calculate the prevalence rate per 1000 eligible women. Segmented regression analysis of interrupted time series with negative binomial regression was used to examine rates for WCBP aged 16–44 years, and by 10‐year age groups. Prevalence ratios (PR) are presented with 95% confidence intervals (CIs).

**Results:**

Among WCBP aged 16–44 years, there was no statistically significant change in the month‐to‐month prevalence ratio in the post‐ compared to pre‐December 2014 intervention period. A significant decline was, however, observed in the post‐, compared to pre‐April 2018 intervention period (PR = 0.998, [95% CIs: 0.996, 1.000]; *p* = 0.029). Among those aged 16–24 years, a significant decreasing trend in the month‐to‐month prevalence ratio was found in the post‐ compared to pre‐December 2014 intervention period (PR = 0.991, [95% CIs: 0.984, 0.998];*p* <0.01). A marginal effect was observed in the post‐ compared to pre‐April 2018 intervention period for those aged 25–34 years (PR = 0.996, [95% CIs: 0.992, 1.000]; *p* = 0.048).

**Conclusion:**

Although no evidence of change was observed following the December 2014 intervention period, a significant decline in the prevalence ratio of valproate use was observed after the 2018 intervention, which may reflect the introduction of the most recent contraindication measures.

**Key Points**

- This study provides insights into valproate utilisation trends among women of childbearing potential (WCBP) aged 16–44 years in Ireland following two separate European Medicines Agency regulatory interventions in December 2014 and April 2018.
- Among WCBP aged 16–44 years, we found no significant change in the trends in valproate use following the 2014 intervention; however, we observed a significant decline in the trends in use of valproate in the post‐, compared to pre‐2018 intervention period.
- The rate of decline observed was greatest among those aged 16–24 years following the first intervention in 2014, and among those aged 25–34 years following the second intervention in 2018.
- We found no significant change in trends (i.e. the slopes of the time series) pre‐ and post‐regulatory intervention in the oldest age group 35–44 years.

**Plain Language Summary**

This study focused on valproate, a medication commonly used to manage epilepsy and bipolar disorder. We examined its use among women of childbearing potential (WCBP) aged 16–44 years in Ireland following the introduction of new prescribing guidance issued in December 2014 and April 2018. We used anonymous monthly national pharmacy dispensing data for Ireland between 1st January 2014 and 31st December 2019 to examine the number of WCBP using valproate before and after each prescribing guidance was issued. Our results showed that the number of WCBP aged 16–44 years dispensed valproate before and after December 2014 was similar. However, the number of WCBP aged 16–44 years using valproate decreased after the second prescribing guidance was issued in April 2018, compared to the number of WCBP using valproate before this time. When we examined the use of valproate for the different age groups, we found that the number of WCBP in the youngest age group (16–24 years) using valproate after December 2014 had decreased compared to the number using valproate before this time point. Collectively, our findings show a decline in valproate use after the 2018 guidance, which may reflect the influence of the most recent prescribing guidance.

## 1 INTRODUCTION

Sodium valproate (Epilim®) is a broad‐spectrum anti‐epileptic drug (AED) licenced for the management of epilepsy and bipolar disorder in all EU countries, and in certain member states for migraine prophylaxis.^1^ For some patients, such as those with idiopathic generalised epilepsy, valproate is recognised as the best treatment option.^2^, ^3^ Similarly, although its efficacy as a mood stabiliser is comparable to lithium,^4^ valproate is reported to be commonly prescribed in women of childbearing potential (WCBP) with bipolar disorder.^5^ When used during pregnancy, however, valproate is associated with a risk of major congenital malformations.^6^, ^7^ Moreover, recent evidence shows that in‐utero exposure to valproate is also associated with behavioural teratogenesis, or negative neurodevelopmental outcomes.^8^, ^9^, ^10^

Following an EU‐wide safety review of valproate by the European Medicines Agency's (EMA) Pharmacovigilance Risk Assessment Committee (PRAC) in 2014, warnings on the use of valproate medicines in women and girls were strengthened due to the risk of malformations and developmental problems in babies exposed to valproate in‐utero.^11^ These updated warnings, which were formally communicated to healthcare professionals (HCPs) in all EU countries through a Direct Healthcare Professional Communication (DHPC) issued in November/December 2014, aimed to improve HCP and patient awareness of the teratogenic risks associated with valproate, and ultimately to reduce the prescribing of valproate in WCBP when alternative treatments are available.^11^ Educational materials directed at HCPs and patients were also implemented to further support risk communication. In 2016, concerns were raised that these restrictions had not been sufficiently effective.^12^ This prompted a further EU‐wide valproate safety review^13^ in March 2017, which resulted in more stringent measures relating to the use of valproate in pregnancy and in WCBP, including the implementation of a pregnancy prevention programme (PPP). These measures, which were communicated to HCPs through a second DHPC in April 2018,^14^, ^15^ included a contraindication on the use of valproate for bipolar disorder or prophylaxis of migraine attacks during pregnancy; a contraindication on the use of valproate for epilepsy during pregnancy unless there is no suitable alternative treatment; and a contraindication on the use of valproate in any WCBP unless the conditions of the new PPP are fulfilled.^13^ Educational materials were also updated to reflect these new measures. Since December 2014, the regulator of medicines in Ireland, the Health Products Regulatory Authority (HPRA), issued or approved a series of communications^16^ to HCPs to highlight the teratogenic risk associated with valproate use during pregnancy, and to communicate the updated recommendations for use of valproate in this patient population. A timeline of all regulatory interventions at European and national level between 2014 and 2019 is presented in Figure S1.

Previous European studies indicate a declining trend in valproate use among WCBP over time,^17^, ^18^, ^19^ and since the 2014 EMA regulatory intervention.^20^, ^21^, ^22^ To date, however, little is known about the impact of the EMA restrictions on valproate use among WCBP in Ireland. Therefore, the aim of this drug utilisation study was to examine trends in valproate use among WCBP aged 16–44 years in Ireland between 2014–2019 using national pharmacy claims data, and to evaluate the effect of the recent EMA regulatory interventions (December 2014 and April 2018) on the rate of valproate use among WCBP pre‐ and post‐intervention using segmented regression analysis of interrupted time series.

## 2 METHODS

The STrengthening the Reporting of Observational Studies in Epidemiology (STROBE) guidelines were used in the reporting of this study.^23^

### 2.1 Study design

A repeated cross‐sectional study with interrupted time series analysis (ITSA) was performed. ITSA is a robust, quasi‐experimental approach used to evaluate the longitudinal effects of an intervention on a specified outcome over‐time, where the intervention is expected to “interrupt” the level and/or trend of the outcome, following its introduction.^24^ National pharmacy claims data from Ireland was used to examine changes in valproate use among WCBP after two specified regulatory events in time—the December 2014 DHPC and the April 2018 DHPC. In Ireland, the average age of mothers at maternity in 2017 was 32.8 years; with the majority (99.4%) aged 16–44 years.^25^ In the present study, WCBP were defined as those aged 16–44 years, and in accordance with the definition of WCBP included in the valproate pregnancy prevention programme guide for HCPs (i.e., a pre‐menopausal female who is capable of becoming pregnant).^26^

### 2.2 Data source

This study used individual‐level national pharmacy claims data from the Health Service Executive‐Primary Care Reimbursement Service (HSE‐PCRS) in Ireland. Detailed information about the service has been previously outlined.^27^ Briefly, the HSE‐PCRS is a national pharmacy claims database, which funds three main community drug schemes: General Medical Services (GMS) scheme, Drugs Payment Scheme (DPS), and the Long Term Illness (LTI) scheme. In Ireland, valproate prescriptions for persons with epilepsy are dispensed free of charge under the LTI scheme^27^; while valproate dispensed under the GMS and DPS schemes is subject to a subsidy co‐payment, and is more likely to reflect use of this drug for other indications (e.g. bipolar disorder).^19^ Currently, epilepsy is the only condition on the LTI scheme for which valproate is conventionally prescribed in patients aged ≥16 years. Therefore, it can be assumed that data from the LTI scheme provide an accurate summary of the national dispensing pattern of valproate for epilepsy alone.^27^ All prescriptions recorded in the HSE‐PCRS are coded using the World Health Organisation (WHO) Anatomical Therapeutic Chemical (ATC) classification system, and anonymised patient demographic details, including age and gender, are also available. Pharmacy claims data, at the individual‐level, from these three community drug schemes was used to identify valproate (ATC code: N03AG01) prescriptions dispensed for women aged 16–44 years overall, and by 10‐year age groups (16–24, 25–34 and 35–44 years), from community pharmacies in Ireland between 1st January 2014 and 31st December 2019, inclusive.

### 2.3 Statistical analysis

**FIGURE 1:**
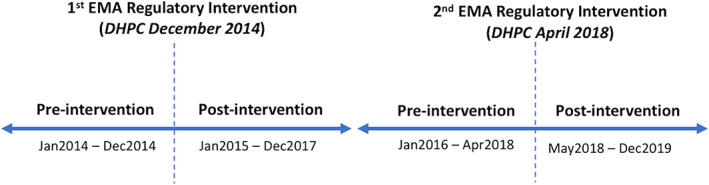
Timeline for segmented regression analysis according to the first and second EMA regulatory interventions. DHPC, direct healthcare professional communication; EMA, European Medicines Agency

Prevalence measures used to report study findings are defined in Box 1. Segmented regression of ITSA was performed using HSE‐PCRS national pharmacy claims data to examine trend (slope) changes in the month‐to‐month prevalence ratios of valproate use before and after both EMA regulatory interventions in December 2014 and April 2018 (i.e. the two time points when prescribers and pharmacists received a DHPC informing them of EMA regulatory changes). Among WCBP, we examined the rate of valproate use overall across all three community drug schemes. Given the different patient populations eligible for reimbursement of valproate under the three community drug schemes, in the results we also present the rate of valproate use in this population at the level of individual drug scheme, so that individuals in the LTI Scheme (i.e. persons with a diagnosis of epilepsy) are distinguished from those with other conditions. Level changes (i.e. an immediate change in rate following the intervention) were not examined, as an immediate change in valproate claims was not anticipated at the time of each intervention. The primary outcome was the change (trend change) in the rate per 1000 eligible women of monthly valproate use post EMA regulatory intervention (DHPC) in December 2014 and April 2018 compared to the time‐periods preceding these time points (baseline trend) as shown in Figure 1. For monthly time series data, at least 12 data points before and after the intervention are recommended.^24^ For the post‐intervention period following the first EMA intervention in December 2014, we chose a time period, which did not include the April 2018 EMA regulatory intervention arising from the second EU‐wide safety review triggered in March 2017 (Figure S1). For the second EMA intervention in April 2018, a pre‐intervention period from 1 year after the first EMA intervention until the second intervention in April 2018, was chosen (i.e. January 2016–April 2018). Annual population estimates, by females and age bands 16–24, 25–34 and 35–44 years, from the Central Statistics Office (CSO) for Ireland for the years 2014–2019, were used to calculate the prevalence rate of valproate use per 1000 eligible women (www.cso.ie). Due to overdispersion of the data, we used negative binomial regression to examine the rates (with an offset term for the population denominator) for the overall population (16–44 years). To examine the change per month in the prevalence rate post‐ compared with pre‐intervention, prevalence ratios with 95% confidence intervals (CIs) are reported. The prevalence ratios estimate the change per month in the prevalence rate of valproate use in the post‐ compared with pre‐intervention time period, and were calculated by exponentiating the regression (beta) coefficients from the regression analysis. For the first intervention, the prevalence ratio for the “baseline trend” was defined as the time period between January 2014 and December 2014; for the second intervention, the prevalence ratio for the “baseline trend” was defined as the time period between January 2016 and April 2018. We also stratified the data by 10‐year age groups and conducted stratified segmented regression models for those aged 16–24, 25–34, and 35–44 years. All data analyses were performed using Stata v16 and significance at *p* <0.05 is assumed.

BOX 1 **Prevalence rate (prevalence)**

The prevalence rate refers to the number of WCBP dispensed valproate for each month divided by the total population of women in the specified age group for the same month. This rate is multiplied by 1000 to give the rate per 1000 eligible population.

**Prevalence ratio (month‐to‐month prevalence ratio)**

The prevalence ratios reported refer to the average change per month in the ratio of the prevalence rate of WCBP dispensed valproate, that is the “month‐to‐month prevalence ratio”. For the post‐intervention period, the prevalence ratio demonstrates the additional effect of the intervention on the prevalence rate (as a ratio of the average month‐to‐month change) compared to that of the pre‐intervention period. The prevalence ratios are calculated by exponentiating the regression (beta) coefficients for the pre‐intervention and post‐intervention effects from the regression analysis.

## 3 RESULTS

### 3.1 Study population

At the start of the study period in January 2014, national pharmacy claims data revealed that *n* = 1993 women aged 16–44 years in Ireland were dispensed valproate. This included *n* = 391 (20.2%) aged 16–24 years, *n* = 561 (29.0%) aged 25–34 years, and *n* = 1041 (53.9%) aged 35–44 years. By December 2019, the number of women aged 16–44 years in Ireland dispensed valproate had declined to *n* = 1569. This decrease was found across all age groups (*n* = 261 (16.6%) aged 16–24 years, *n* = 421 (26.8%) aged 25–34 years, and *n* = 887 (56.5%) aged 35–44 years).

### 3.2 Impact of the EMA intervention overall across the study population aged 16–44 years

**FIGURE 2:**
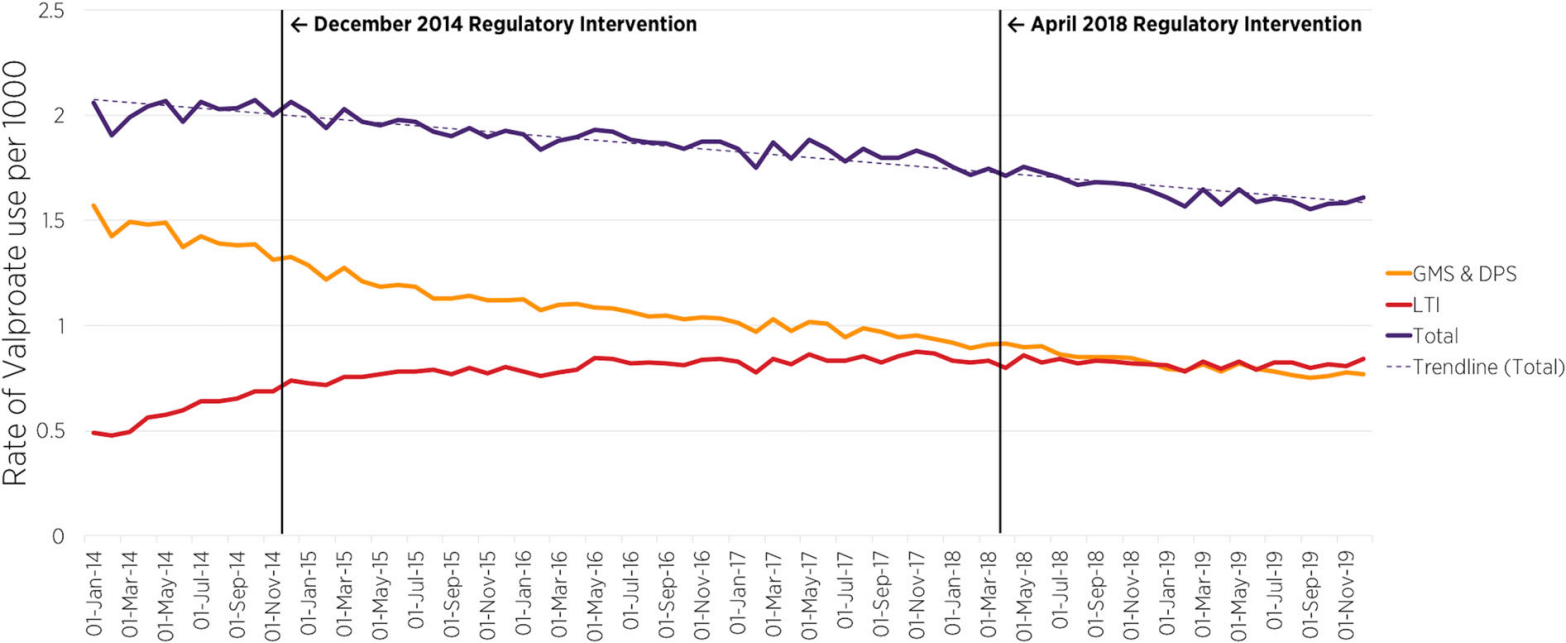
Prevalence per 1000 WCBP aged 16–44 years of valproate use overall (GMS, DPS, and LTI) and by community drug scheme between 2014 and 2019, and a time series interruption where the 2014 and 2018 European Medicines Agency regulatory intervention is indicated by the vertical black line. DPS, drugs payment scheme; GMS, general medical services; LTI, long‐term illness

**FIGURE 3:**
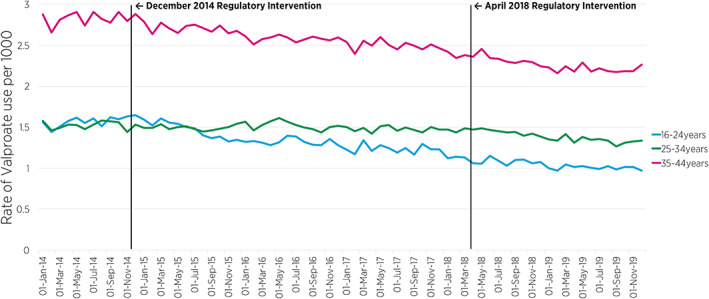
Overall (GMS, DPS, and LTI) prevalence per 1000 of women of childbearing potential using valproate (by 10‐year age groups) between 2014 and 2019, and a time series interruption where the 2014 and 2018 European Medicines Agency regulatory intervention is indicated by the vertical black line. DPS, drugs payment scheme; GMS, general medical services; LTI, long‐term illness

Figure 2 presents the prevalence rate of valproate use (by community drug scheme) per 1000 women aged 16–44 years and shows the valproate use trends pre‐post the first (December 2014) and pre‐post the second (April 2018) EMA regulatory interventions. A time series interruption where the 2014 and 2018 EMA regulatory intervention occurred is indicated by the vertical line in Figures 2 and 3. Overall, across the three community drug schemes, prior to the implementation of any intervention, the rate of valproate use among women aged 16–44 years between January 2014 (2.06 per 1000) and December 2014 (2.06 per 1000) was stable. However, following the EMA regulatory intervention in December 2014 and April 2018, there was a decrease in the overall rate in subsequent months from December 2014, that continued across the time‐period of the second EMA intervention, to December 2019 (1.61 per 1000, 95% CI: 1.53, 1.69) (Figure 2). Segmented regression of ITSA of the post‐ compared to pre‐December 2014 intervention showed no significant change (PR = 0.997; [95% CIs: 0.994, 1.000]); however, there was a statistically significant decline in the prevalence ratio in the post‐, compared to pre‐April 2018 intervention period (PR = 0.998; [95% CIs: 0.996, 1.000], *p* = 0.029) (Table 1).

**TABLE 1:** Prevalence ratio of valproate use and 95% CIs overall and by 10‐year age groups, for baseline trend and trend changes per month pre and post two regulatory interventions in December 2014 and April 2018

	Pre‐December 2014 (baseline trend per month)		Post‐December 2014 (trend change per month)		Pre‐April 2018 (baseline trend per month)		Post‐April 2018 (trend change per month)	
	PR	95% CI	PR	95% CI	PR	95% CI	PR	95% CI
Overall	0.999	0.997, 1.002	0.997	0.994, 1.000	0.996	0.995, 0.997	0.998*	0.996, 1.000
Age group (years)								
16–24	1.001	0.995, 1.007	0.991*	0.984, 0.998	0.993	0.991, 0.995	0.999	0.994, 1.004
25–34	1.000	0.995, 1.005	1.000	0.994, 1.005	0.998	0.996, 1.000	0.996*	0.992, 1.000
35–44	0.998	0.995, 1.002	0.998	0.994, 1.003	0.996	0.995, 0.998	0.998	0.995, 1.001

Abbreviations: CI, confidence interval; PR, prevalence ratio.

* *p* <0.05.

### 3.3 Impact of the EMA intervention across 10‐year age groups

Figure 3 shows the prevalence rate of valproate use across the three community drug schemes, by 10‐year age groups. Segmented regression of ITSA revealed a significant decreasing trend (slope) in the month‐to‐month prevalence ratio of valproate use among those aged 16–24 years in the post‐December 2014 intervention period (PR = 0.991, 95% CIs: [0.984, 0.998], *p* = 0.009) compared to the pre‐December 2014 intervention period (Table 1). The analysis also shows a decreasing trend in the post‐April 2018 intervention period compared to the pre‐April 2018 intervention period, though not statistically significant. Among women aged 25–34 years, there was also a marginally significant decline in the month‐to‐month prevalence ratio of valproate use in the post‐April 2018 intervention period (PR = 0.996, 95% CI: [0.992, 1.000], *p* = 0.048) compared to the pre‐April 2018 intervention period. The month‐to‐month prevalence ratio of valproate use in the oldest age group (35–44 years) remained relatively stable across the two intervention periods (Table 1).

## 4 DISCUSSION

### 4.1 Summary of main findings

We found a decline in use of valproate among WCBP aged 16–44 years, following the implementation of targeted regulatory interventions in December 2014 and April 2018. The rate of decline observed was greatest among those aged 16–24 years following the first intervention in December 2014, and among those aged 25–34 years following the second intervention in April 2018. The declining trend in valproate use among women aged 16–24 years, might suggest that, during the study observation period, valproate was not being initiated in WCBP, where alternative treatment options were available. The decline in the rate of valproate use among women aged 25–34 years seen after the April 2018 regulatory intervention might indicate cases of successful discontinuation, or indeed a decline in initiation of valproate in this age group. There was no change in the use of valproate among women aged 35–44 years following either the December 2014 or April 2018 regulatory intervention, which suggests these interventions did not affect patterns of valproate use in this age group.

### 4.2 Comparison with previous studies

Our findings are similar to a recently published drug utilisation study conducted in Estonia, which reported a non‐significant decline in the rate of valproate prescribing in women of childbearing age (15–44 years) following the 2014 EMA intervention, and a significant decline among females aged 15–19 years.^28^ The decline among 16–24 year olds in our study may indicate that the 2014 intervention reduced first‐time prescribing of valproate among neurologists in Ireland. These patterns observed, support our recent survey study findings from the Irish HCP setting, which found that all responding neurologists reported that they had considered an alternative treatment before initiating their patient on valproate.^29^ In addition, a recent drug utilisation study examined valproate prescribing patterns in five European countries (France, Germany, Spain, Sweden and the UK) and found that, in all countries, the proportion of WCBP prescribed valproate had decreased (between 30.1% and 52.3%) following the 2014 intervention.^30^ Similarly, our study identified an overall decline in the month‐to‐month use of valproate among WCBP (16–44 years) following the 2014 EMA intervention.

To date, the findings from valproate utilisation studies from other European countries have revealed differences in the impact of the 2014 intervention at the individual‐country level. In Lithuania, a valproate utilisation study reported a general decrease in valproate for females under 50 years, but failed to report any significant impact of the 2014 EMA restrictions other than a delayed decrease in use among girls under 15 years.^31^ A drug utilisation study conducted in Stockholm examined the impact of the 2014 EMA regulatory intervention on initiations of valproate, and reported a significant decline among women aged ≤45 years with a psychiatric disorder, but not epilepsy.^21^ In contrast, the recent Estonian utilisation study reported the decline in prescribing following the 2014 intervention to be among neurologists, and not psychiatrists.^28^ However, another large cohort study of valproate prescribing patterns among 5.4 million women in three European countries (France, Italy, and the UK), found that the incidence of valproate prescribing decreased in women of childbearing age for both bipolar disorders and epilepsy, following the 2014 EMA recommendations.^22^ The study also reported the overall decline in incident prescribing of valproate between 2007 and 2016 to be greatest among those aged 30–39 years and >40 years. The utilisation study conducted in Estonia found limited evidence to support a decline in prevalence in the older age groups (35–39 and 40–44 years).^28^ Our study, similarly found stable trends in valproate use among women aged 35–44 years following the 2014 intervention. Indeed, this may reflect long‐standing use of valproate in this older age group, where successful discontinuation may be less easily achieved.

### 4.3 Strengths and limitations

This is the first study to examine the potential impact of both the 2014 and 2018 EMA regulatory interventions on the prevalence rate of valproate use among WCBP, aged 16–44 years in Ireland. Moreover, to the authors' knowledge, we are not aware of other published literature, which has examined the potential influence of the most recent 2018 EMA regulatory intervention on valproate utilisation trends at the population‐level. An additional strength of this study is that the pharmacy dispensing data used was derived from a large national population‐based pharmacy claims database. The findings from this study are, therefore, representative of the general population of women aged 16–44 years, in Ireland who received valproate during the study observation period. Our study examined pharmacy claims data over a long time‐period (2014–2019), overall and at individual drug‐scheme level. In addition, this study used ITSA, which is a well‐described quasi‐experimental design approach to estimate the effects of an intervention in non‐randomised settings.^24^ However, this study has limitations that must be considered. Firstly, a limitation of ITSA includes “history bias”, as other external factors, not accounted for or examined as part of this study, may have affected the utilisation patterns of valproate use during the study observation period.^32^ In addition, since two specific regulatory interventions occurred during the time‐series examined in this study, it is difficult to examine the pre‐2018 period of the second intervention in isolation of the post‐2014 intervention period. Our findings should, therefore, be interpreted with caution, since we cannot assume causality. Second, this study is limited by the number of data points (months of pharmacy claims data). Indeed, increasing the number of data points would increase the precision of our estimates of the slope (trend) changes in the month‐to‐month prevalence ratios of valproate use in this population. In addition, this study is limited to the data available from the LTI, GMS and DPS, therefore, we are unable to account for valproate dispensed outside of these community drug schemes; however, given the economic incentive of the community drug schemes examined in the present study, it is expected that most patients would be participating in one of these schemes. We also did not have access to information on co‐dispensed contraceptive use in the population examined, though a previous study conducted in Ireland prior to the introduction of the PPP, reports the rate of concomitant contraceptive in WCBP dispensed valproate under the GMS scheme to be low.^19^ Further, we had no information on an individual's adherence to the PPP, which would be important to consider in instances where valproate utilisation has remained unchanged (e.g. 35–44 years cohort). Although valproate prescriptions dispensed under the LTI scheme can be considered to be for epilepsy, the indication for which valproate was dispensed under the GMS and DPS schemes is not precisely known. In addition, prior to December 2013, to enhance the cost‐effective provision of medicines in Ireland, patients with GMS eligibility could not also benefit from LTI eligibility. As of 1st December 2013, however, patients with both GMS and LTI eligibility can now access medicines for their specific condition, free of charge, under the LTI scheme.^33^ This has resulted in an administrative change in how medicines for such patients are dispensed, whereby patients who previously had their LTI‐related medicines dispensed under the GMS scheme for a co‐payment fee, can now have these medicines dispensed free‐of‐charge under the LTI scheme, not requiring a co‐payment. Therefore, this may have contributed to the early temporal growth (Figure 2) in 2014 in the prevalence of females receiving valproate under the LTI scheme. Finally, as it was not possible to determine prescribers' specialisation within each drug scheme, it is difficult to establish with precision where information dissemination and education should be targeted to support optimised use of valproate in this patient population.

### 4.4 Implications

This drug utilisation study provides an important insight into patterns of valproate use among WCBP for both national and European regulators and policy makers. In Ireland, there is evidence of a decline in the use of this teratogenic drug among WCBP, particularly among those aged 16–24 and 25–34 years, in line with the timeline of the introduction of the EMA regulatory interventions in 2014 and 2018. However, a decline in trends was not observed across all relevant age groups. Further research to examine trends in valproate use among the older (35–44 years) cohort of WCBP, including the indication for use and reasons for continued use, would provide a better understanding of these utilisation patterns. It is possible that WCBP who continued to receive valproate during the study observation period did so in accordance with the 2014 and 2018 EMA guidance (i.e. other treatments were ineffective or not tolerated and the conditions of the PPP were fulfilled), however the present study did not have access to the information required to fully investigate this hypothesis. Indeed, access to registry data for WCBP prescribed valproate, would provide utility here. Efforts should therefore be made to establish such a population‐based register in Ireland. Further research that evaluates adherence to the PPP, and underlying indication‐based patterns of valproate prescribing, is needed to determine the overall impact of these interventions on valproate use.

### 4.5 Conclusion

The 2014 regulatory intervention was followed by a decline in the use of valproate among WCBP in Ireland, significantly among those aged 16–24 years. By comparison, the 2018 regulatory intervention was followed by a significant decline in the use of valproate among WCBP, particularly among women aged 25–34 years in Ireland. This may be related to the introduction of new contraindication measures, particularly in WCBP unless the conditions of the PPP are fulfilled. Further research may be required to determine adherence to regulatory advice across all age groups. Linkage of data between relevant clinical healthcare and dispensing databases, as has been achieved in other areas of pharmacoepidemiology research in Ireland,^27^ could support such research in this area as well as ongoing monitoring of effectiveness of the PPP nationally. Continued efforts may be required to ensure that all prescribers and their patients are aware of the current guidance and restrictions on the use of valproate in WCBP, in particular the most recent 2018 EMA restrictions.

## CONFLICT OF INTEREST

The authors declare no conflict of interest.

## AUTHOR CONTRIBUTIONS

Kathleen Bennett, Maeve Mullooly, Niamh Buckley, Yvonne Looney (and Almath Spooner) were involved in the concept and design of the study. Kathleen Bennett obtained data from the HSE‐PCRS. John E. Hughes, Kathleen Bennett, and Maeve Mullooly conducted analysis and led on the interpretation of the data. The manuscript was prepared by John E. Hughes, Maeve Mullooly, and Kathleen Bennett. Niamh Buckley, Sinead Curran, and Yvonne Looney and all authors reviewed all drafts of the manuscript. All authors read and approved the final manuscript.

## ETHICS STATEMENT

This study used anonymised aggregated pharmacy claims data, provided by the HSE‐PCRS for the purpose of this analysis, and therefore ethical approval was not required.

## Supporting information


**Supplementary Figure1** Timeline of Regulatory Interventions implemented to strengthen warnings on the use of valproate medicines in women & girls (2013–2014 EMA referral procedure) and New measures to avoid valproate exposure in pregnancy (2017–2018 EMA referral procedure). PRAC: Pharmacovigilance Risk Assessment Committee; CMDh: Coordination Group for Mutual Recognition and Decentralised Procedures – Human; HPRA: Health Products Regulatory Authority Ireland; MIMS: Monthly Index of Medical Specialities; ANSM: Agence Nationale de Sécurité du Médicament et des Produits de Santé
