# Supplementary material for: Valproate utilisation trends among women of childbearing potential in Ireland between 2014 and 2019: A drug utilisation study using interrupted time series
Source: Pharmacoepidemiol Drug Saf. 2022 Mar 31;31(6):661–9. doi: 10.1002/pds.5427 (PMC9315025; doi:10.1002/pds.5427)
Supplement: Supplementary file 1 — Supplementary Figure1 Timeline of Regulatory Interventions implemented to strengthen warnings on the use of valproate medicines in women & girls (2013–2014 EMA referral procedure) and New measures to avoid valproate exposure in pregnancy (2017–2018 EMA referral procedure). PRAC: Pharmacovigilance Risk Assessment Committee; CMDh: Coordination Group for Mutual Recognition and Decentralised Procedures – Human; HPRA: Health Products Regulatory Authority Ireland; MIMS: Monthly Index of Medical Specialities; ANSM: Agence Nationale de Sécurité du Médicament et des Produits de Santé [file PDS-31-661-s001.docx]

**Electronic Supplementary Material 1**

**Article title:** Valproate utilisation trends among women of child-bearing potential in Ireland between 2014 and 2019: a drug utilisation study using interrupted time series

**Journal name:** Pharmacoepidemiology and Drug Safety

**Authors:** John E Hughes^1^, Niamh Buckley^2^, Yvonne Looney^2^, Sinead Curran^2^, Maeve Mullooly^1^, Kathleen Bennett^1,3^

**Affiliations and institutions:**

^1^Division of Population Health Sciences, Royal College of Surgeons in Ireland, Dublin, Ireland.

^2^Health Products Regulatory Authority, Earlsfort Terrace, Dublin 2, Ireland.

^3^RCSI Data Science Centre, Beaux Lane House, Mercer Street Lower, Dublin 2, Ireland.

**Corresponding author:**

Kathleen Bennett,

Data Science Centre, Division of Population Health Sciences, Royal College of Surgeons in Ireland, Dublin, Ireland.

Email: [kathleenebennett@rcsi.ie](mailto:kathleenebennett@rcsi.ie)

ORCID: 0000-0002-2861-7665

**
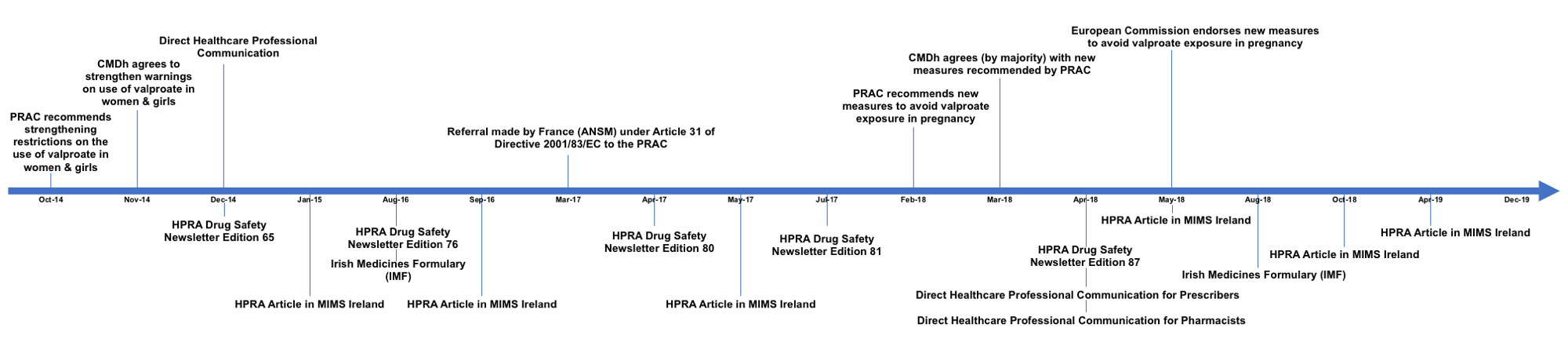
**

Supplementary Figure1 Timeline of Regulatory Interventions implemented to strengthen warnings on the use of valproate medicines in women & girls (2013 – 2014 EMA referral procedure) and New measures to avoid valproate exposure in pregnancy (2017-2018 EMA referral procedure). PRAC: Pharmacovigilance Risk Assessment Committee; CMDh: Coordination Group for Mutual Recognition and Decentralised Procedures – Human; HPRA: Health Products Regulatory Authority Ireland; MIMS: Monthly Index of Medical Specialities; ANSM: Agence Nationale de Sécurité du Médicament et des Produits de Santé
